# Image Enhancement via Subimage Histogram Equalization Based on Mean and Variance

**DOI:** 10.1155/2017/6029892

**Published:** 2017-12-18

**Authors:** Liyun Zhuang, Yepeng Guan

**Affiliations:** ^1^School of Communication and Information Engineering, Shanghai University, Shanghai, China; ^2^Faculty of Electronic and Information Engineering, Huaiyin Institute of Technology, Huai'an, China; ^3^Key Laboratory of Advanced Displays and System Application, Ministry of Education, Shanghai, China

## Abstract

This paper puts forward a novel image enhancement method via Mean and Variance based Subimage Histogram Equalization (MVSIHE), which effectively increases the contrast of the input image with brightness and details well preserved compared with some other methods based on histogram equalization (HE). Firstly, the histogram of input image is divided into four segments based on the mean and variance of luminance component, and the histogram bins of each segment are modified and equalized, respectively. Secondly, the result is obtained via the concatenation of the processed subhistograms. Lastly, the normalization method is deployed on intensity levels, and the integration of the processed image with the input image is performed. 100 benchmark images from a public image database named CVG-UGR-Database are used for comparison with other state-of-the-art methods. The experiment results show that the algorithm can not only enhance image information effectively but also well preserve brightness and details of the original image.

## 1. Introduction

Enhancement technology is regarded as one of the most active fields of digital image processing. It improves the quality and appearance for low contrast image, and it can be used in monitoring, imaging systems, human-computer interaction [1–3], and many other areas [4–9]. The histogram equalization (HE) technique is simple and easily implemented, which is most extensively utilized for contrast enhancement. HE utilizes the cumulative density function (CDF) of image for transferring the gray levels of original image to the levels of enhanced image. The main drawback of HE is that it tends to change the mean brightness of the image to the middle level of the dynamic range and results in annoying artifacts and intensity saturation effects. This drawback makes HE technique unsuitable for many consumer electronics applications, for example, TV and cameras.

In order to overcome the shortcomings mentioned above, many other HE-based methods have been proposed, such as the brightness preserving bihistogram equalization (BBHE) [10], dualistic subimage histogram equalization (DSIHE) [11], and minimum mean brightness error bihistogram equalization (MMBEBHE) [12]. BBHE [10] partitions the histogram based on the image mean while DSIHE [11] uses image median to segment. MMBEBHE [12] recursively divides the image histogram into multiple groups based on mean brightness error (MBE). Although these methods have made great progress, they still have their own drawbacks, including failing with images having nonsymmetric distribution [10], failing to preserve mean brightness [11], producing more annoying side effects [12], and losing structural information [13]. In these techniques, however, desired improvement may not always be achieved, and the difference between input and output image is minimal [14].

Chen and Ramli proposed the method called recursive mean-separate histogram equalization (RMSHE [15]), in which the authors suggested recursive division of histograms based on the local mean. The mean brightness of processed image approaches towards the mean brightness of input image. Wang et al. improved DSIHE [11] into recursive subimage histogram equalization (RSIHE [16]) based on contrast enhancement, by introducing recursive segmentation in the similar manner as Chen and Ramli proposed in [15], although this method is similar to RMSHE [15] but it uses median values instead of mean values to divide histogram into subhistograms.

Adaptively modified histogram equalization (AMHE) [17] method is developed by Kim et al., which can modify the probability density function (PDF) of the grayscale as well as apply histogram specification to the modified PDF. Unfortunately, the entire redistribution to the original histogram by those methods can cause overenhancement, underenhancement, and some artifacts appearing in some smooth regions. Although the AMHE [17] does not produce any degradation, it darkens the bright areas of the sky and fails to boost the brightness of the dark regions.

In addition, some other methods based on histogram equalization for contrast enhancement with brightness enhancement have also been proposed, such as the dynamic histogram specification introduced by Sun et al., which preserves the shape of the input image histogram but does not enhance it significantly [18]. Tsai et al. suggested a contrast enhancement algorithm for color images [19, 20]. Huang et al. proposed an adaptive gamma correction with weighting distribution (AGCWD [21]) to enhance the contrast and preserve the overall brightness of an image; in the method, the gamma correction and a probability distribution for luminance pixels were used. The AGCWD technique may not give desired results when an input image lacks bright pixels since the highest intensity in the output image is bounded by the maximum intensity of the input image, because the highest enhanced intensity will never cross the maximum intensity of the input image [22]. Besides, AGCWD [21] leads to loss of information in processed image due to its sharp increasing resultant transformation curve described below.

An image enhancement technique using the idea of exposure value, named image enhancement using exposure-based subimage histogram equalization (ESIHE [23]), was advanced. The method divided the clipped histogram into two parts by using the precalculated exposure threshold [24]. The effects of using intensity exposure in histogram segmentation before histogram clipping were studied in [25]. Through simulation on standard images, low contrast images, and noisy images, the study showed that [25] could yield a certain enhancement results; however, the method usually causes underenhancement. Tang and Mat Isa introduced an algorithm named bihistogram equalization using modified histogram bins (BHEMHB) [26], which segmented the input histogram based on the median brightness and altered the histogram bins before HE is applied, but it made limited improvement for contrast.

In order to effectively increase the contras of the input image with brightness and details well preserved, an efficient algorithm named Mean and Variance based Subimage Histogram Equalization (MVSIHE) is developed in this paper. The proposed method is more effective for preserving the mean brightness and details of the enhanced image while improving the contrast compared with some other state-of-the-art methods. According to the experiments based on 100 images for our method, we know that the MVSIHE technique can achieve the multiple objectives of entropy maximization, details, and brightness preservation as well as control on overenhancement. The main contributions of this paper are as follows. Firstly, we introduce the mean and variance based algorithm to divide the histogram of the image. Secondly, a novel transformation called hyperbolic tangent transformation is developed to modify the histogram bins to overcome this domination problem. Thirdly, we put forward a normalization transformation, which can make the brightness component of the output image have a wider dynamic range and the output image look more natural and clearer. Furthermore, results indicate that the proposed method is a better approach compared to the state-of-the-art methods.

This paper is organized as follows: Section 2 describes the proposed MVSIHE method. Data samples and performance evaluations are given in Section 3.  Section 4 shows experimental results and comparisons with state-of-the-art methods, and our concluding remarks are included in Section 5.

## 2. Proposed Image Enhancement Method

### 2.1. Threshold Calculation Based on Mean and Variance

The histogram of an image is divided into four parts with three thresholds which are adaptive and obtained by the same method. The procedure to obtain the thresholds will be presented in detail as follows.

An input image *X* is given; let *H*  [*l*_low_, *l*_up_] be the global histogram of the input image *X*, where *l*_low_ and *l*_up_ represent lower and uppermost intensities of the image *X*. *H*(*l*) is the histogram of the gray level *l*, which is described as

(1) $$\begin{array}{c} H\left(\;l\;\right)=n_{l}\;for\;\;l=l_{low},\ldots ,l_{up}, \end{array}$$

where *n*_*l*_ is the of gray level *l* in the image *X*, the pdf of the image, pdf(*l*), can be defined as

(2) $$\begin{array}{c} pdf\left(\;l\;\right)=\frac{H\left(l\right)}{\left(M*N\right)}\;for\;\;l=l_{low},\ldots ,l_{up}, \end{array}$$

where *M∗N* is the total number of pixels in the input image *X*.

The threshold value for histogram segmentation can be obtained. First, we divide the whole histogram into two parts by an adaptive threshold *k*. Then the two parts can be presented as Sub_0_{0 ~ *k*} and Sub_1_{*k* + 1 ~ *l*_max_}. The probability of each part can be solved by

(3) Sub1,2:  ω0=∑l=0kpdfl,Sub2,2:  ω1=∑l=k+1lmaxpdfl.

Next, the mean value of each part can be calculated by

(4) Sub1,2:  μ0=∑l=0kl∗pdflω0,Sub2,2:  μ1=∑l=k+1lmaxl∗pdflω1.

Therefore, the mean of the whole image *X* is described as

(5) $$\begin{array}{c} \mu =\mu_{0}\omega_{0}+\mu_{1}\omega_{1}. \end{array}$$

Then, we can seize the variance between the two parts by

(6) $$\begin{array}{c} \sigma^{2}\left(\;k\;\right)=\omega_{0}{\left(\;\mu_{0}-\mu \;\right)}^{2}+\omega_{1}{\left(\;\mu_{1}-\mu \;\right)}^{2}. \end{array}$$

Then the optimization model can be defined as

(7) $$\begin{array}{c} \underset{k}{\max}\;\sigma^{2}\left(\;k\;\right). \end{array}$$

We can obtain the optimal threshold *k*_opt_ by (7), which is utilized to segment the histogram of image. Note that we set *k*_2_ = *k*_opt_; the optimal thresholds *k*_1_ and *k*_3_ of the two parts up and down the threshold *k*_2_ can also be obtained in the same way as the above, respectively. Finally, the histogram *H*  [*l*_low_, *l*_up_] is segmented into four subhistograms; that is,

(8) $$\begin{array}{c} H\left[\;l_{low},l_{up}\;\right]=\overset{4}{\underset{i=1}{⋃}}{sub}^{i,4}\left[\;l_{low}^{i,4},l_{up}^{i,4}\;\right], \end{array}$$

*l*_low_^*i*,4^ and *l*_up_^*i*,4^ are the boundary values of the luminance range within the *i*th segmentation. Hence, the all subimages are captured by

(9) $$\begin{array}{c} {sub}^{i,4}=\left\{\;l\left(\;i,j\;\right)\;|\;{l^{i,4}}_{low}\leq l\left(\;i,j\;\right)\leq {l^{i,4}}_{up},\;\forall \left(\;i,j\;\right)\in l\;\right\}, \end{array}$$

the pdf of *i*th subhistogram is represented by

(10) $$\begin{array}{c} {pdf}_{{sub}^{i,4}}=\frac{h_{{sub}^{i,4}}\left(l\right)}{n_{{sub}^{i,4}}}\;for\;\;l={l^{i,4}}_{low},\ldots ,{l^{i,4}}_{up}, \end{array}$$

where *n* is the number of pixels of the *i*th segmentation. After the segment of input image histogram, the next stage of processing procedure is histogram modification. As mentioned in the introduction, CHE emphasizes the domination of high-frequency histogram bins, thus resulting in loss of details in the image. Low-frequency histogram bins tend to be swallowed by high-frequency bins in the neighborhood. MVSIHE modifies the histogram bins to overcome this domination problem. Histogram bin modification is performed using (11) for the subhistogram [27].

(11) new_pdfsubi,4=epdfsubi,4−e−pdfsubi,4epdfsubi,4+e−pdfsubi,4for  l=li,4low,…,li,4up,

where *n*_sub^*i*,4^_ is the total number of pixels in the *i*th subimage.

(12) cdfsubi,4l=∑l=llowlnew_pdfsubi,4lfor  l=li,4low,…,lupi,4.

### 2.2. Histogram Equalization

CHE involves mapping an input gray level *L* using transformation function *f*(*l*), which can be defined as

(13) $$\begin{array}{c} f\left(\;l\;\right)=X_{0}+\left(\;X_{0}-X_{L-1}\;\right)*cdf\left(\;l\;\right), \end{array}$$

where *X*_0_ and *X*_*L*−1_ represent the minimum and maximum gray levels, respectively. As observed in (8), the remapping of the input image is within the entire dynamic range [*X*_0_, *X*_*L*−1_] after applying CHE. The proposed method equalizes the modified subhistograms by (14); thereafter, the equalized subhistograms are integrated to produce the final enhanced output image.

(14) flsubi,4=li,4low+li,4up−li,4low∗cdfsubi,4lfor  l=li,4low,…,li,4up.

### 2.3. Normalization of Intensity Levels

In our proposed method, each segment is equalized independently and output image is obtained by adding the equalized subsegments. This may result in saturation of intensities and interference caused by nonuniform light; in order to solve the problems, we utilize the normalization of intensity levels of the processed image. The normalization transformation is defined as

(15) $$\begin{array}{c} T\left(\;L\;\right)=\frac{L-l_{min}}{L_{max}-L_{min}}\left(\;L_{up}-L_{low}\;\right)+L_{low}, \end{array}$$

where *L* is a matrix of the input image's luminance component and *L*_max_ and *L*_min_ are the maximum and the minimum values of *L*, respectively. *L*_low_ and *L*_up_ are the boundary values of the luminance range within [*L*_low_, *L*_up_], without loss of generality, *L*_low_ is set as 0, and *L*_up_ is 255 to obtain a maximum luminance range for 256 gray levels.

**Figure 1:**
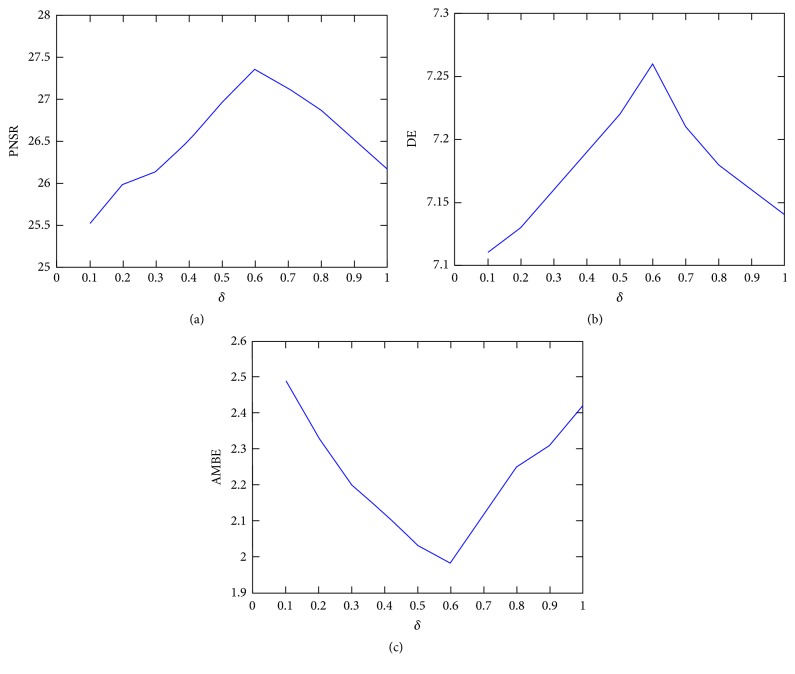
Parameter analysis and comparison. (a) Variation of PNSR with different *δ*. (b) Variation of AMBE with different *δ*. (c) Variation of DE with different *δ*.

After normalization of intensity levels, for the sake of getting a more comprehensive and informative information output image, we fuse INT_img_ and INP_img_ together by the following:

(16) $$\begin{array}{c} PRC_{img}=\delta *INT_{img}+\left(\;1-\delta \;\right)*INP_{img}, \end{array}$$

where INT_img_ is image obtained after applying (15), INP_img_ is input image, and PRC_img_ is finally output image. Parameter is between 0 and 1.  Figure 1 shows the statistical results (100 test images) with different parameters *δ*. From Figures 1(a), 1(b), and 1(c), we can know that the average values of Peak Signal-to-Noise Ratio (PSNR), Discrete Entropy (DE), and Absolute Mean Brightness Error (AMBE) can obtain optimum value when *δ* is roughly to 0.6.

## 3. Data Samples and Performance Evaluations

### 3.1. Data Samples

In this paper, we compare the performance of the proposed method with some other state-of-the-art methods: DSIHE [11], RMSHE [15], MMBEBHE [12], RSIHE [16], ESIHE [23], and BHEMHB [26]. The MVSIHE and other HE-based image enhancement methods for comparison are tested by using 100 benchmark images from a public image database named CVG-UGR-Database [28].

### 3.2. Subjective Evaluation

Subjective evaluation of contrast enhancement is necessary as well as objective evaluation. The enhancement results can only be appreciated if the resultant image gives pleasurable effect in appearance. By visual quality inspection the judgment of annoying artifacts, overenhancement, and unnatural enhancement can be done. The visual assessment results are effective quality measures to judge the performance of contrast enhancement algorithm.

### 3.3. Objective Evaluation

Qualitative analysis involves visually evaluating the image enhancement results. The quality of the enhanced images determines the capability of the techniques, which are justified by human eyes. Here, a qualitative analysis regarding the amount of details of the image, level of contrast, homogeneity of regions, and naturalness is performed; we can establish numerical justifications by quantitative measurements. However, it is difficult to find an objective measure that is in accordance with the subjective assessment due to the lack of any universally accepted criterion. Here, we evaluate the performance of enhancement techniques using three quality metrics: Peak Signal-to-Noise Ratio (PSNR), Discrete Entropy (DE), and Absolute Mean Brightness Error (AMBE).

#### 3.3.1. Evaluation of Contrast Enhancement

The proposed method not only enhances the contrast of the image, but also obtains a natural-looking output image without undesirable artifacts. The noise level should not be amplified during the enhancement process [29]. For this reason, two analyses named PSNR and image contrast function are used. To calculate the PSNR value, MSE is firstly computed by (18). PSNR is broadly used to evaluate the quality achievement between the original and output images [13, 30–33] and the degree of contrast enhancement in the image. A large PSNR value which is desired for it means that the processed image is least degraded compared with the original input image.

(17) MSE=1MN∑m∑nXm,n−Ym,n2,

(18) PSNR=10 log10⁡L−12MSE,

where *X*  (*m*, *n*) is the gray level of the original image at a 2D position (*m*, *n*) and *Y*  (*m*, *n*) is the gray level of the processed image at the same position.

Besides PSNR, image contrast function is used to evaluate the contrast improvement as well, as indicated in (20) [34, 35].

(19) Ccontrast=1MN∑m=1M ∑n=1NY2m,n−1MN∑m=1M ∑n=1NYm,n2,

where *M* and *N* represent the width and height of the image, respectively. The greater *C*_contrast_, the greater dynamic range of gray levels; thus the output image can provide better contrast and additional information contained in the image. *C*_contrast_ is then taken as a logarithm to convert it into decibel (dB) unit by

(20) $$\begin{array}{c} {C^{*}}_{contrast}=10\;\log_{10}C_{contrast}. \end{array}$$

#### 3.3.2. Evaluation of the Richness of Information

Entropy is a measure of the richness of information in the image, and the larger entropy value the image has, the higher the information contained in the output image is.

The entropy for the whole image can be defined by

(21) $$\begin{array}{c} DE=\overset{L-1}{\underset{l=0}{\sum }}e\left(\;l\;\right)=-\overset{L-1}{\underset{l=0}{\sum }}p\left(\;l\;\right)\log_{2}p\left(\;l\;\right). \end{array}$$

The entropy of the image can achieve maximum value only when *p*(0) = *p*(1) = ⋯ = *p*(*L* − 1) = 1/*L* [27]. This is the scenario when the probability distribution of the image intensity values is uniform, which is the concept behind HE.

#### 3.3.3. Evaluation of Brightness Preservation

AMBE is usually used to measure mean brightness preservation, which can be mathematically represented by (22) [36–38]. AMBE exhibits the difference in mean brightness between the input and the output image. Mean brightness of the input and processed images is calculated using (23) and (24), respectively. Thus, a small AMBE value is desired, and a zero AMBE value is the best result.

(22) AMBE=EX−EY,

(23) EX=1MN∑m ∑nXm,n,

(24) EY=1MN∑m ∑nYm,n,

where *E*(*X*) and *E*(*Y*) are the mean brightness of the input and processed images, respectively.

## 4. Experiment Results and Discussion

### 4.1. Experiment Results

In this section, the simulation results of the proposed method MVSIHE are compared with existing histogram equalization based methods mentioned.  Table 1 provides the list of methods with their detailed description. The comparison is from the aspects of contrast enhancement, brightness preservation, naturalness of the image, and ability to preserve details in the image.

**Figure 2:**
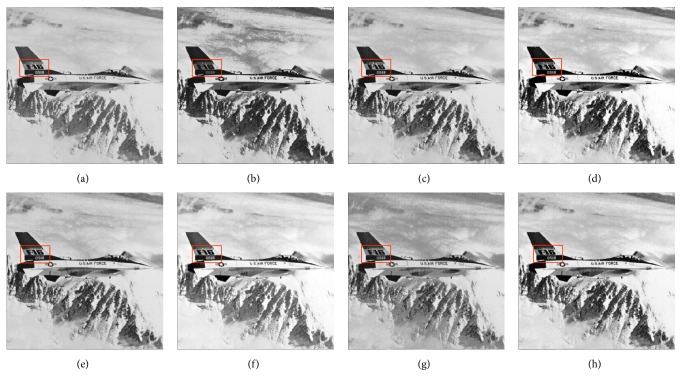
Results for F16. (a) Original image. (b) DSIHE. (c) RMSHE. (d) MMBEBHE. (e) RSIHE. (f) ESIHE. (g) BHEBHD. (h) Proposed MVSIHE.

**Figure 3:**
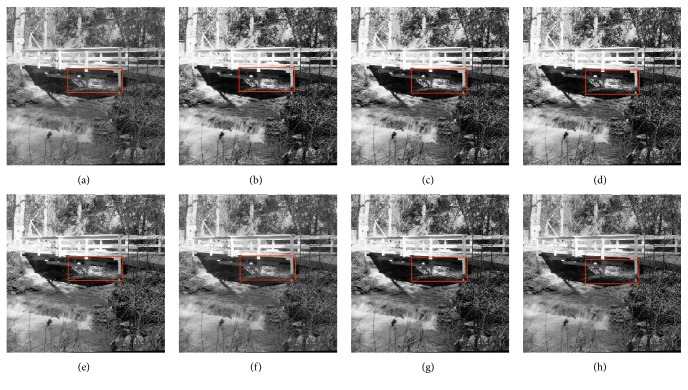
Results for Bridge. (a) Original image. (b) DSIHE. (c) RMSHE. (d) MMBEBHE. (e) RSIHE. (f) ESIHE. (g) BHEBHD. (h) Proposed MVSIHE.

**Figure 4:**
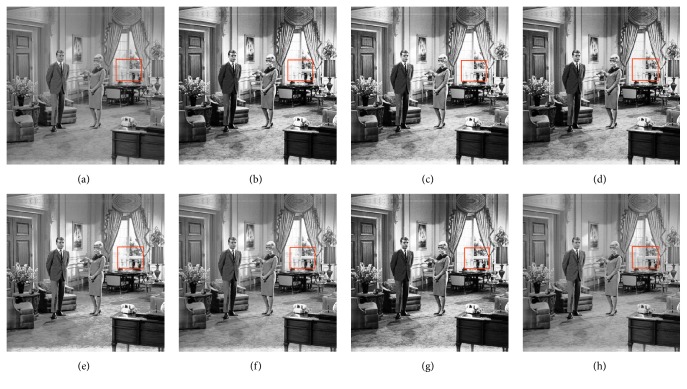
Results for Couple. (a) Original image. (b) DSIHE. (c) RMSHE. (d) MMBEBHE. (e) RSIHE. (f) ESIHE. (g) BHEBHD. (h) Proposed MVSIHE.

**Figure 5:**
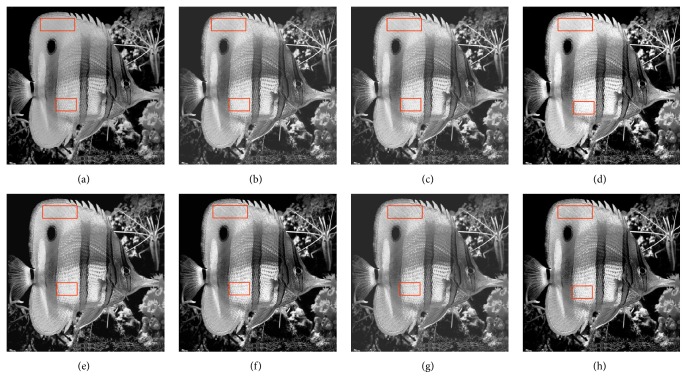
Results for Fish. (a) Original image. (b) DSIHE. (c) RMSHE. (d) MMBEBHE. (e) RSIHE. (f) ESIHE. (g) BHEBHD. (h) Proposed MVSIHE.

**Figure 6:**
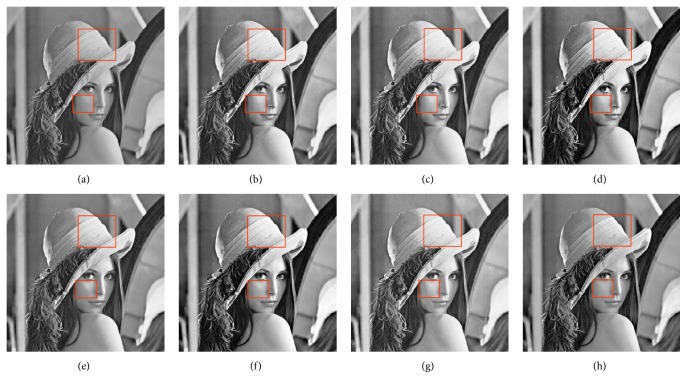
Results for Lena. (a) Original image. (b) DSIHE. (c) RMSHE. (d) MMBEBHE. (e) RSIHE. (f) ESIHE. (g) BHEBHD. (h) Proposed MVSIHE.

**Figure 7:**
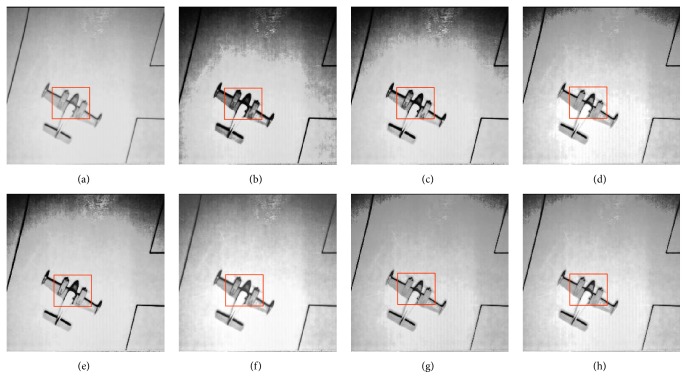
Results for Plane. (a) Original image. (b) DSIHE. (c) RMSHE. (d) MMBEBHE. (e) RSIHE. (f) ESIHE. (g) BHEBHD. (h) Proposed MVSIHE.

In this paper, the test images are given names as F16, Bridge, Couple, Fish, Lena, and Plane; they are presented in this study for initial performance evaluation on the proposed MSVIHE. The results obtained for each image are presented in Figures 2–7, respectively. Image (a) indicates the input image, while images (b) to (i) represent the respective resultant images after applying other compared methods and the proposed MSVIHE. The quantitative results of these test images are illustrated in Tables 2–5. The best value for each analysis is in bold face.

For the first test image F16 in Figure 2(a), the proposed MVSIHE yields output image with the mean brightness closest to the input image. The overall appearance of the image is very similar to the input image and for the proposed MVSIHE method can get the lowest AMBE value. The proposed method can well preserve most of the details of the image compared with the other methods for it grapes the highest value of entropy. This can be seen from the highlighted area with red boxes. MVSIHE also produces images with homogeneous texture. Most of the image area, particularly the background of the image, appears to have a smooth texture with a few small regions. The largest PSNR value is obtained by the MVSIHE-ed image, which shows that the technique least amplified the noise level in the image during the enhancement process. The proposed MVSIHE can well preserve the brightness of the processed image due to its largest contrast value.

For the test image Bridge in Figure 3(a), just as the contrast enhancement which is more significant compared with the other techniques, most details of the image are well preserved with its highest value of entropy. This can be seen from the words highlighted with red boxes. Processed images are with relatively good contrast, the value of the contrast by MVSIHE is ranked as second, and the effects of contrast enhancement are not far-off between all the methods. The MVSIHE method least amplifies the noise level in the image during the enhancement process for it can obtain the largest PSNR value.

The proposed MVSIHE can simultaneously enhance the overall contrast of the test image Couple to an optimum level and preserve the details of the image, which can be observed on the window area highlighted with a box, as shown in Figure 4(h). It is clear that the saturation effect is less apparent and thus the window area can be clearly seen. This saturation effect (i.e., the window area regions become too bright) can be observed in the RSIHE-ed image. Observation on the ability of the proposed MVSIHE to preserve details is supported by the entropy measurement, in which the enhanced image has an entropy value larger than most of the methods, indicating that the information entropy is well preserved. The MVSIHE-ed image has the largest value of PSNR (i.e., 22.6008), showing that BHEMHB least degrades the image during the enhancement process. In addition, the MVSIHE-ed image has the largest contrast measurement, which suggests that the proposed method can well preserve the brightness of the output image.

The MVSIHE-ed image has the largest value of PSNR (i.e., 26.401), showing that MVSIHE least degrades the image during the enhancement process. The proposed MVSIHE can simultaneously enhance the overall contrast of the Fish image to an optimum level and preserve the details. This outcome can be observed on the fish scale highlighted with a box, as in Figure 5(h). Observation on the ability of the proposed MVSIHE grapes the biggest value of entropy, demonstrating that the information entropy is well preserved. The ability to preserve details comes with a small tolerance in mean brightness preservation. Furthermore, the effects of contrast enhancement are less momentous for all methods, which demonstrates relatively good contrast. The ability of MVSIHE in contrast enhancement is about the same to the other methods. The output image enhanced by MVSIHE, as shown in Figure 5(h), also exhibits a natural look, which means that it does not look too artistic after the enhancement process.

The input image Lena has the characteristics that regions that are either fully black or fully white are relatively few, as shown in Figure 6(a). The resultant image enhanced with the proposed MVSIHE has a clearer contour compared with images using the other methods, as can be seen on regions within boxes. Unlike images enhanced with other techniques, especially RMSHE, the image enhanced with MVSIHE presented fewer saturation effects. The proposed MVSIHE ranked first place for test image Lena in the entropy measurement, a ranking that is slightly less than that of other methods. We can know that MVSIHE is specifically designed to preserve the details; the difference reveals that the performance of MVSIHE is comparable with others in retaining image details. Furthermore, the proposed method can well preserve brightness for its lowest AMBE value.

For the test image Plane in Figure 7(a), the proposed MVSIHE produces an output image with most of the details well preserved because it possesses the highest entropy value. This result can be seen on regions highlighted with boxes, where the writing does not disappear and small details, such as edges of the plane, can be seen. The shifting effect of mean brightness is pregnant in the DSIHE-ed and RMSHE-ed images, resulting in the loss of naturalness in these images. By contrast, the resultant image enhanced with MVSIHE has a smooth texture, wherein less nonhomogenous regions are observed, especially on the background, compared with other techniques. In addition, the MVSIHE-ed image has the largest contrast measurement.

**Figure 8:**
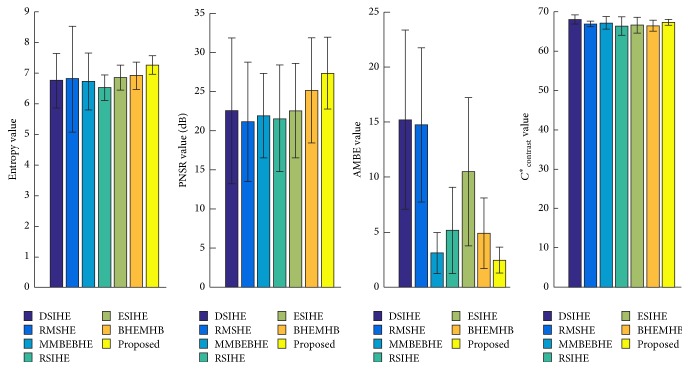
Average values and standard deviations of quantitative analyses for 100 test images.

Findings on the performance of the proposed techniques for the six test images, namely, F16, Fish, Plane, and Lena, are satisfactory when compared with those of the seven other methods. Thus, apart from these six test images, the four objective evaluation functions (i.e., entropy, PSNR, AMBE, and Contrast) are employed on the 100 test images to further validate the capability and performance of the proposed MVSIHE.  Figure 8 presents the average values of these quantitative analyses for 100 test images.

Figure 8 indicates that the proposed method illustrates excellent performance when compared with the other HE-based methods. In average, the MVSIHE-ed image contains the highest amount of information. It can well preserve the richness and details of information in output image due to its highest entropy value, which reaches 7.26 for an average of 100 test images. The proposed MVSIHE outperforms all the other methods, with its largest PSNR value, which shows that the output images enhanced by MVSIHE have a natural appearance with minimum artifacts compared with others. The proposed method can least degrade the image during the enhancement process.

### 4.2. Discussions

With regard to mean brightness, the MVSIHE-ed image demonstrates high capability, especially when compared with the DSIHE-ed images. DSIHE method yields an image that is too bright when referred to the original image. AMBE values for all the techniques are computed, and MVSIHE can obtain the lowest value compared to all the others. The naturalness of the image is maintained in the MVSIHE-ed image, because the image is enhanced at a sufficient level without introducing an unpleasant look or nonhomogeneous regions while improving the contrast of the input image. The highest PSNR value by the MVSIHE-ed image indicates that MVSIHE enhances the image with minimum noise and artifacts. Contrast measurements show that the MVSIHE can get the largest value mostly, which illustrates that the contrast enhancement performance of MVSIHE is better than others.

Moreover, MVSIHE acquires the lowest AMBE value. The AMBE value obtained by MVSIHE is notably better than that obtained by RSIHE technique because RSIHE is specifically designed to maintain the mean brightness of the image. The lowest AMBE value indicates that MVSIHE possesses the highest capability in retaining the mean brightness of the image compared with all the other methods, in which the output images enhanced with MVSIHE typically have a mean brightness closest to the input image.

With regard to the overall contrast enhancement, otherwise, the proposed MVSIHE ranked second among the seven methods. The range of contrast measurements is small (i.e., only 1.69 dB), which indicates that despite its outstanding performance in detail preservation and mean brightness preservation, MVSIHE demonstrates comparable performance in contrast enhancement. Both qualitative and quantitative analyses show that the proposed MVSIHE yields promising enhancement results.

## 5. Conclusion

This paper presents a new method referred to as the Mean and Variance based Subimage Histogram Equalization (MVSIHE) with brightness and details preservation. The main idea lies on recursively separating the input histogram based on the mean and variance. The effect of intensity levels normalization and fusion strategy is also investigated in this paper. Unpleasant artifacts and unnatural enhancement may occur due to excessive equalization while enhancing the contrast of an input image, and the ultimate goal of MVSIHE is to allow higher level of brightness and details preservation as much as possible. The contrast of the input image is effectively increased with brightness and details well preserved. All findings are supported by experimental results, which have shown that the proposed method has superior performance to some state-of-the-art methods. In the future, the proposed MVSIHE could be modified so that it can be incorporated in several application areas such as digital photography, video processing, and other applications in consumer electronics.

## Figures and Tables

**Table 1:** Properties of the proposed MVSIHE and other HE-based methods.

Methods	Implementation steps^a^	Main focus
DSIHE	(1) HS using probability density function	(1) Contrast enhancement
	(2) HE	(2) Detail preservation

RMSHE	(1) HS using mean brightness (*γ* = 2)	(1) Mean brightness preservation
	(2) HC using the middle gray level	(2) Detail preservation
	(3) HE	

MMBEBHE	(1) HS using minimum mean brightness error	(1) Mean brightness reservation
	(2) HE	

RSIHE	(1) HS using median brightness (*γ* = 2)	(1) Mean brightness preservation
	(2) HE	

ESIHE	(1) HC using the average number of intensity occurrence	(1) Mean brightness preservation
	(2) HS using exposure threshold	(2) Enhancement rate restriction

BHEMHB	(1) HS using median brightness (*γ* = 1)	(1) Mean brightness preservation
	(2) Modification of histogram bins	(2) Detail preservation
	(3) HE	

MVSIHE	(1) HS using mean and variance brightness (*γ* = 2)	(1) Mean brightness preservation
	(2) Modification of histogram bins	(2) Detail preservation
	(3) HE	(3) Contrast enhancement
	(4) Fuse processed image with input image	

^a^HS indicates histogram segmentation, HC indicates histogram clipping, and HE indicates histogram equalization.

**Table 2:** PSNR calculated for the test images.

Image name	DSIHE	RMSHE	MMBEBHE	RSIHE	ESIHE	BHEMHB	Proposed
F16	20.9870	21.8760	24.4849	22.1130	22.8690	23.9420	**27.0944**
Bridge	24.2212	22.9133	23.5086	24.3100	28.4320	26.7650	**31.4561**
Couple	19.7178	18.3277	21.0514	20.5600	21.6780	20.4635	**22.6008**
Fish	20.1459	19.7673	22.0862	24.9780	25.4060	26.4010	**28.2385**
Lena	23.5663	22.1785	22.7730	24.8000	25.7990	26.5950	**29.3948**
Plane	28.7635	27.6540	28.3268	17.3390	18.2420	20.6130	**30.3730**

**Table 3:** DE calculated for the test images.

Image name	DSIHE	RMSHE	MMBEBHE	RSIHE	ESIHE	BHEMHB	Proposed
F16	6.3590	6.0909	6.5023	6.4910	6.6120	6.6660	**7.3898**
Bridge	7.2512	7.1588	7.0540	6.5257	7.3680	7.1456	**7.8408**
Couple	6.9940	**7.9891**	6.8467	6.3971	7.4100	7.2350	7.6047
Fish	6.1604	6.1689	6.2672	5.9100	5.9850	6.0170	**7.1066**
Lena	7.1996	7.0085	7.2195	7.4610	7.4970	7.5620	**7.7539**
Plane	6.3447	6.1205	6.2500	6.3050	6.3800	6.4470	**7.1520**

**Table 4:** AMBE calculated for the test images.

Image name	DSIHE	RMSHE	MMBEBHE	RSIHE	ESIHE	BHEMHB	Proposed
F16	20.2554	5.7030	0.4496	6.4810	2.8740	1.3340	**0.1322**
Bridge	2.3752	3.6479	**2.1850**	3.6592	3.7860	2.3750	3.5623
Couple	4.0264	11.9858	0.7215	5.0718	2.1570	**0.5652**	2.2811
Fish	9.5402	11.7333	8.2674	3.7260	3.8960	4.5760	**3.3199**
Lena	6.0463	10.3377	0.8662	5.1090	2.5310	2.4650	**0.8474**
Plane	16.6670	13.2179	**3.3521**	13.1660	11.2240	8.1610	4.6093

**Table 5:** Contrast calculated for the test images.

Image name	DSIHE	RMSHE	MMBEBHE	RSIHE	ESIHE	BHEMHB	Proposed
F16	69.73	68.86	69.98	68.82	69.28	69.07	**70.15**
Bridge	66.16	66.30	**66.52**	63.32	63.01	65.16	66.43
Couple	64.56	64.65	63.98	62.61	62.01	64.48	**64.67**
Fish	66.13	66.22	67.15	66.85	66.42	**67.60**	67.56
Lena	**64.83**	63.22	64.17	64.33	63.66	64.52	64.62
Plane	70.11	69.67	69.21	69.25	69.33	70.06	**70.24**

## References

[1] Du S., Ward R. K. (2010). Adaptive region-based image enhancement method for robust face recognition under variable illumination conditions. *IEEE Transactions on Circuits and Systems for Video Technology*.

[2] Sun G., Liu S., Wang W., Chen Z. (2014). Dynamic range compression and detail enhancement algorithm for infrared image. *Applied Optics*.

[3] Huang T.-H., Shih K.-T., Yeh S.-L., Chen H. H. (2013). Enhancement of backlight-scaled images. *IEEE Transactions on Image Processing*.

[4] Iqbal M. Z., Ghafoor A., Siddiqui A. M. (2013). Satellite image resolution enhancement using dual-tree complex wavelet transform and nonlocal means. *IEEE Geoscience and Remote Sensing Letters*.

[5] Riaz M. M., Ghafoor A., Sreeram V. Fuzzy C-means and principal component analysis based GPR image enhancement.

[6] Roller W., Berger A., Szentes D. Technology based training for radar image interpreters.

[7] Casaca W., Boaventura M., De Almeida M. P., Nonato L. G. (2014). Combining anisotropic diffusion, transport equation and texture synthesis for inpainting textured images. *Pattern Recognition Letters*.

[8] Chang C.-C., Wu B.-R., Hsu H.-J., Liang J.-W., Peng Y.-C., Tai W.-K. Texture synthesis approach using cooperative features.

[9] Ahire R. B., Patil V. S. Overview of satellite image resolution enhancement techniques.

[10] Kim Y.-T. (1997). Contrast enhancement using brightness preserving bi-histogram equalization. *IEEE Transactions on Consumer Electronics*.

[11] Wang Y., Chen Q., Zhang B. (1999). Image enhancement based on equal area dualistic sub-image histogram equalization method. *IEEE Transactions on Consumer Electronics*.

[12] Chen S.-D., Ramli A. R. (2003). Minimum mean brightness error bi-histogram equalization in contrast enhancement. *IEEE Transactions on Consumer Electronics*.

[13] Kim M., Chung M. G. (2008). Recursively separated and weighted histogram equalization for brightness preservation and contrast enhancement. *IEEE Transactions on Consumer Electronics*.

[14] Wang C., Ye Z. (2005). Brightness preserving histogram equalization with maximum entropy: a variational perspective. *IEEE Transactions on Consumer Electronics*.

[15] Chen S.-D., Ramli A. R. (2003). Contrast enhancement using recursive mean-separate histogram equalization for scalable brightness preservation. *IEEE Transactions on Consumer Electronics*.

[16] Sim K. S., Tso C. P., Tan Y. Y. (2007). Recursive sub-image histogram equalization applied to gray scale images. *Pattern Recognition Letters*.

[17] Kim H., Lee J., Lee J., Oh S., Kim W. Contrast Enhancement Using Adaptively Modified Histogram Equalization.

[18] Sun C.-C., Ruan S.-J., Shie M.-C., Pai T.-W. (2005). Dynamic contrast enhancement based on histogram specification. *IEEE Transactions on Consumer Electronics*.

[19] Tsai C.-M., Yeh Z.-M. (2008). Contrast enhancement by automatic and parameter-free piecewise linear transformation for color images. *IEEE Transactions on Consumer Electronics*.

[20] Tsai C.-M., Yeh Z.-M., Wang Y.-F. (2011). Decision tree-based contrast enhancement for various color images. *Machine Vision and Applications*.

[21] Huang S.-C., Cheng F.-C., Chiu Y.-S. (2013). Efficient contrast enhancement using adaptive gamma correction with weighting distribution. *IEEE Transactions on Image Processing*.

[22] Rahman S., Rahman M. M., Hussain K., Khaled S. M., Shoyaib M. Image enhancement in spatial domain: A comprehensive study.

[23] Singh K., Kapoor R. (2014). Image enhancement using exposure based sub image histogram equalization. *Pattern Recognition Letters*.

[24] Hanmandlu M., Verma O. P., Kumar N. K., Kulkarni M. (2009). A novel optimal fuzzy system for color image enhancement using bacterial foraging. *IEEE Transactions on Instrumentation and Measurement*.

[25] Tang J. R., Isa N. A. M. (2016). Intensity exposure-based bi-histogram equalization for image enhancement. *Turkish Journal of Electrical Engineering & Computer Sciences*.

[26] Tang J. R., Mat Isa N. A. (2017). Bi-histogram equalization using modified histogram bins. *Applied Soft Computing*.

[27] Zhu Y., Huang C. (2012). An Adaptive Histogram Equalization Algorithm on the Image Gray Level Mapping. *Physics Procedia*.

[28] CVG-UGR-Database, http://decsai.ugr.es/cvg/dbimagenes

[29] Hasikin K., Isa N. A. M. Fuzzy enhancement for nonuniform illumination of microscopic Sprague Dawley rat sperm image.

[30] Zadbuke A. S. (2012). Brightness preserving image enhancement using modified dualistic sub image histogram equalization. *International Journal of Scientific and Engineering Research*.

[31] Yoo J.-C., Ahn C. W. (2012). Image matching using peak signal-to-noise ratio-based occlusion detection. *IET Image Processing*.

[32] Anand B., Thirugnanam K., Sebastian J. Adaptive display power management for mobile games.

[33] Liang K., Ma Y., Xie Y., Zhou B., Wang R. (2012). A new adaptive contrast enhancement algorithm for infrared images based on double plateaus histogram equalization. *Infrared Physics & Technology*.

[34] Zhang C.-J., Fu M.-Y., Jin M., Zhang Q.-H. (2004). Approach to enhance contrast of infrared image based on wavelet transform. *Hongwai Yu Haomibo Xuebao/Journal of Infrared and Millimeter Waves*.

[35] Tang J. R., Mat Isa N. A. (2014). Adaptive Image Enhancement based on Bi-Histogram Equalization with a clipping limit. *Computers and Electrical Engineering*.

[36] Sengee N., Choi H. (2015). A Novel Filter ed Bi-Histogram Equalization Method. *Journal of Korea Multimedia Society*.

[37] Aedla R., Dwarakish G., Reddy D. V. (2015). Automatic Shoreline Detection and Change Detection Analysis of Netravati-GurpurRivermouth Using Histogram Equalization and Adaptive Thresholding Techniques. *Aquatic Procedia*.

[38] Maurya L., Mahapatra P. K., Kumar A. (2017). A social spider optimized image fusion approach for contrast enhancement and brightness preservation. *Applied Soft Computing*.

